# Raman spectroscopy for agricultural applications

**DOI:** 10.3389/fpls.2025.1607036

**Published:** 2025-09-01

**Authors:** Isaac D. Juárez, Dmitry Kurouski

**Affiliations:** ^1^ Department of Biochemistry and Biophysics, Texas A&M University, College, Station, TX, United States; ^2^ Interdisciplinary Faculty of Toxicology, Texas A&M University, College, Station, TX, United States

**Keywords:** Anova, peak shifting, response characterization, PLS-DA, 2D-COS

## Abstract

Over the last decade, we observe a significant interest in Raman spectroscopy expressed by plant biologists and other agriculturalists. However, many of these people have limited experience in Raman analyses. To this end, we wrote an extensive method-focused manuscript in which we critically discuss key steps in analyses for spectroscopic data. We also conveyed the major differences between analysis of certain peaks vs the full spectra, as well as discussed how both types of spectral analyses could complement each other. We also focused on chemometric analysis of data, including supervised methods, such as PLS-DA. Using our own data, we demonstrated a contribution of spectral preprocessing, model parameters and validation in the performance of supervised methods in data classification. We also critically discussed practical applications of peak fitting and 2-D correlation spectroscopy and demonstrated how these approaches can be useful to extract valuable information about biological processes that take place in plants.

## 1 Introduction

The current future of agriculture lies at the intersection of science, automation, and sustainability. With the human population continuing to grow, crop production must increase by an estimated 70–100% by 2050 to meet demands (Tian et al., 2021). Similarly, as developing countries industrialize and wages rise, global meat consumption has more than quadrupled since the 1960s (Ritchie et al., 2019; Biscarra-Bellio et al., 2023). Addressing these challenges requires scientific and technological innovation, and as such, the field of digital agriculture (DA) has emerged as the focal point. DA combines precision agriculture and automated data-collection tools to optimize agricultural practices and improve productivity (Balasundram et al., 2023). Among these technologies, Raman spectroscopy (RS) has emerged as a vital tool, providing rapid, non-destructive, non-invasive, and highly accurate biochemical analysis of plant and animal systems. These features have made RS critically valuable in early disease detection and the monitoring of biological health.

In crop production, many applications of RS include disease diagnostics, plant phenotyping, and nutrient content monitoring (Payne and Kurouski, 2021; Weng et al., 2021; Juárez and Kurouski, 2024). In animal systems, RS is used for disease diagnostics, dietary monitoring, and quality/safety assessment of animal products such as milk and honey (Shi et al., 2021; Sotiropoulou et al., 2021; Yu et al., 2021; Nilghaz et al., 2022; Tamošiūnas et al., 2022; Juárez et al., 2025; Zhang et al., 2025). For researchers and agriculturalists, understanding the full range of biological insights that can be extracted from Raman spectra is critical to effective usage of this technology. It should be noted that in addition to RS, near-infrared spectroscopy, RGB imaging, fluorescence spectroscopy, hyperspectral imaging, polarization spectroscopy, photoacoustic spectroscopy, and terahertz spectroscopy can be used in the field for disease diagnostics and quality/safety assessment of animal products (Payne and Kurouski, 2021; Weng et al., 2021; Juárez and Kurouski, 2024). More detailed discussion of advantages and disadvantages of those techniques relative to RS are discussed in our recent reviews ( (Farber et al., 2019; Payne and Kurouski, 2021; Juárez and Kurouski, 2024).

When designing Raman-based studies, experiments typically fall into two categories: general comparisons and perturbation-dependent comparisons. General comparison studies focus on evaluating how spectra differ between distinct independent variables, such as comparing the average spectra of crops affected by various nutritional deficiencies. Since these comparisons do not involve any continuous perturbation, the statistical emphasis in these experiments is on identifying unique spectral fingerprints associated with each independent variable. Perturbation-dependent comparisons focus on how a single spectral feature responds to scaled alterations in an independent variable. These studies focus on more complex statistics to track changes in the spectral fingerprint as functions of the perturbation. Both types of studies provide valuable information, but their difference in experimental design necessitates distinct statistical methods.

Statistical methods for analyzing Raman data can be broadly divided based on their interpretation of the spectra. The first type, discrete peak analyses, focuses on individual Raman peaks. The simplest statistical approach involves comparing peak heights between experimental groups and performing a statistical hypothesis test to compare group means. Since Raman peak heights are proportional to the relative abundance of specific biomolecular classes, these methods provide key insights into the biochemical differences between experimental groups. In perturbation-dependent comparisons, additional aspects such as peak shifting and peak response characterization can offer valuable information about molecular structure changes occurring under the perturbation.

The second type, full spectrum analyses, considers the entire Raman spectrum instead of individual peaks. Machine learning algorithms (MLAs) are dominant in these analyses due to their capacity to extract subtle patterns from high dimensionality data (Ruiz-Perez et al., 2020). Supervised MLAs, in particular, can be trained on spectral datasets to build highly accurate classification models for agricultural applications like disease diagnostics. A complementary, although underutilized technique for full spectrum analysis is two-dimensional correlation spectroscopy (2D-COS). 2D-COS can identify which peaks change in response to a perturbation as well as the sequence of said changes, offering mechanistic insights into a biological system’s response to the perturbation (Lasch and Noda, 2019).

In this article, we review methods for analyzing Raman spectral data collected in agricultural experiments to maximize biological insights. Data processing methods typically include three steps: the first step is denoising, the second step is feature extraction, and the third step is modeling. We cover both discrete peak and full-spectrum analyses, discussing how findings from each approach complement the other. These statistical methods are demonstrated using both published and unpublished data primarily from three agricultural experiments: detection of tomato spotted wilt virus (TSWV) isolates in tomatoes, arsenic stress in rice, and aging in mice (Juárez et al., 2024a, 2024b, 2025). Together, these projects encompass both general and perturbation-dependent comparisons, allowing us to highlight the differences in statistical approaches required by different study designs. This article aims to provide new researchers with practical insights in analyzing Raman spectral data effectively.

## 2 Discrete peak analyses

### 2.1 Raman spectra

**Figure 1:**
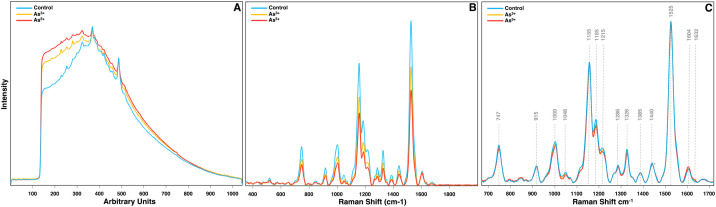
**(A)** Raw, **(B)** baselined, and **(C)** normalized + trimmed Raman spectra of rice leaves from crops grown in water contaminated with As^3+^ and As^5+^. Normalization at the 1440 cm^-1^ peak is indicated with an asterisk.

The first step in analyzing Raman data is plotting the raw spectra for quality control (**Figure 1A**). Since Raman scattering is an inherently weak phenomenon, biological samples often exhibit strong biofluorescence, thus requiring baseline correction such as polynomial fitting and asymmetric least squares. Ideally, baseline correction removes fluorescence and other background noise without distorting peak information, but excessive correction can introduce negative peaks or loss of spectral details. The easiest way to do this is plotting all spectra at once to identify spectra containing artifacts, excessive noise, or obvious outliers should be removed to prevent data skewing. Since RS is a light-based phenomenon, a sample’s color can influence signal strength and quality depending on the laser’s wavelength, causing substantial differences in the raw spectra of otherwise biological similar samples. For example, in the experiment on arsenic stressed rice, chlorotic leaves in the arsenic groups caused the average spectra to be much lower in Raman intensity than the intensity from the control. This would lead to the conclusion that the differences in content are much greater than is true. To mitigate this effect, spectra should always be normalized to a neutral reference peak.

Biological samples also exhibit significant heterogeneity throughout a tissue, so multiple spectra should be collected from each sample to account for variability. Plotting all spectra together helps identify and remove spectra with artifacts, excessive noise, or outliers that could skew results. Additionally, because Raman signal intensity depends on laser wavelength and sample color, variations in pigmentation can drastically affect spectral intensity. For example, in arsenic-stressed rice, chlorotic leaves produced lower Raman intensities than control leaves, potentially exaggerating differences in biochemical content (**Figure 1B**). To correct for these inconsistencies, spectra should be normalized to a stable reference peak. Based on our experience, normalization to the 1440 cm^-1^ peak, which corresponds to CH_2_ bending modes ubiquitous in biological samples, provides the most reliable results (Person and Pimentel, 1953; Farber et al., 2023). Other normalization methods, such as total area normalization, mean centering, and autoscaling, rely on the entire spectrum rather than a neutral reference peak. This can lead to inaccuracies, such as altering the relative differences between experimental groups when comparing their Raman intensity at certain peaks.

**Figure 2:**
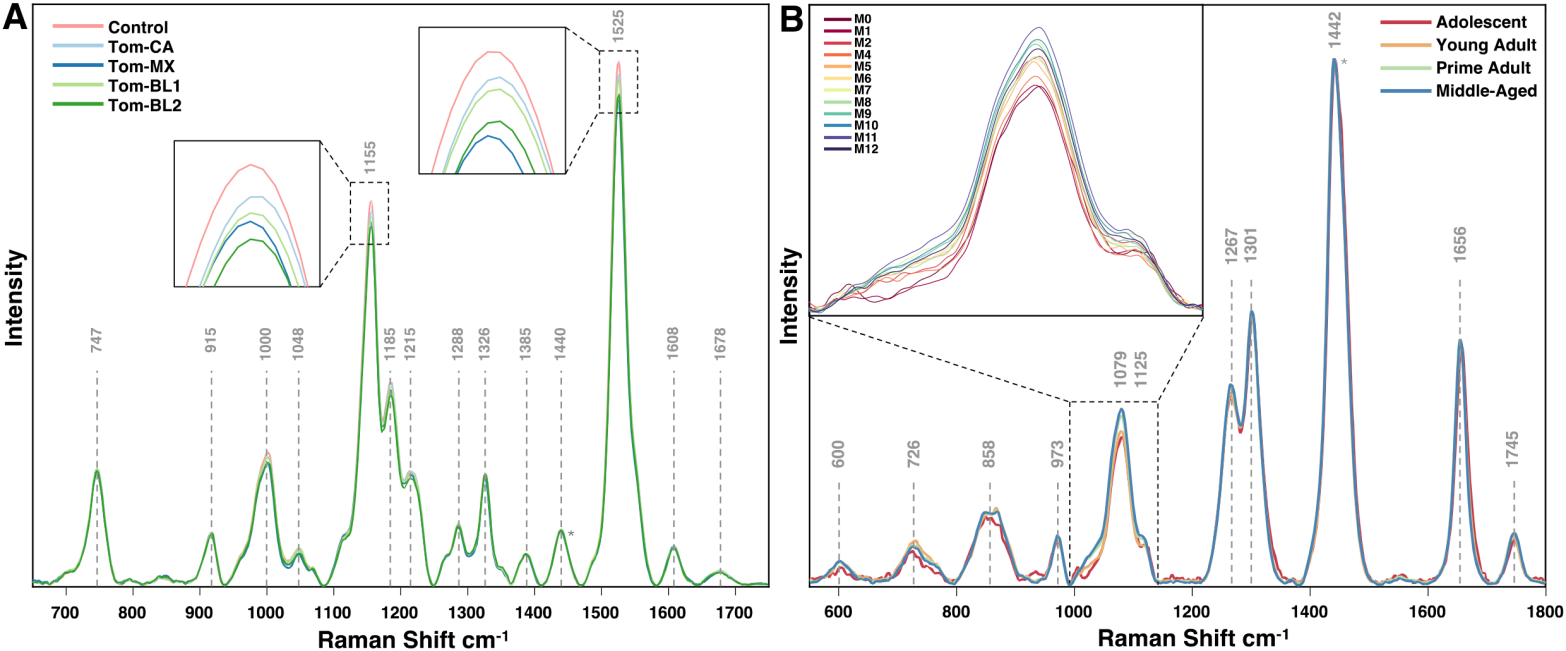
Averaged Raman spectra for **(A)** tomato leaves infected with different isolates of TSWV, and **(B)** mice on vegan diet at different timepoints. Normalization at the 1440 cm^-1^ peak is indicated with an asterisk.

Once spectra are averaged, filtered, and normalized by experimental group, they can be accurately compared for biomolecular content across conditions. For instance, in the experiments discussed here, RS reveals substantial differences in the intensities of the 1155 cm^-1^ and 1525 cm^-1^ peaks in tomato crops infected with TSWV and in rice crops experiencing arsenic stress (**Figures 1C**, **2**). A small increase in peak intensity also exists at the 1604 cm^-1^ peak in arsenic stressed rice. Similarly, in mice study, pronounced spectral variation is observed at the 1079 cm^-1^ peak, which progressively increases with mouse age. Peak assignments for these vibrations indicate decreases in carotenoid content in crops under stress, an increase in phenolic content specifically in arsenic stressed rice, and an increase in lipid content in aging mice.

### 2.2 Analysis of variance

While visual differences in spectral intensity offer valuable insights, statistical testing provides the rigor necessary to draw reliable conclusions. This is especially important when linking changes in specific peak intensity back to differences in sample composition. For analyzing group differences at individual peaks, the two most common tests are one-way ANOVA and the Kruskal-Wallis test, each with distinct assumptions. ANOVA requires normally distributed data, while Kruskal-Wallis is the non-parametric equivalent (Kruskal and Wallis, 1952). However, these tests can only indicate whether one group’s intensity is statistically different from the rest, so *post-hoc* tests are required to identify which specific groups differ. For Anova, Tukey’s HSD (honestly significant difference) test is appropriate when equal variances are assumed (Abdi and Williams, 2010). For Kruskal-Wallis, Dunn’s test is the corresponding *post-hoc* test (Dinno, 2015).

**Figure 3:**
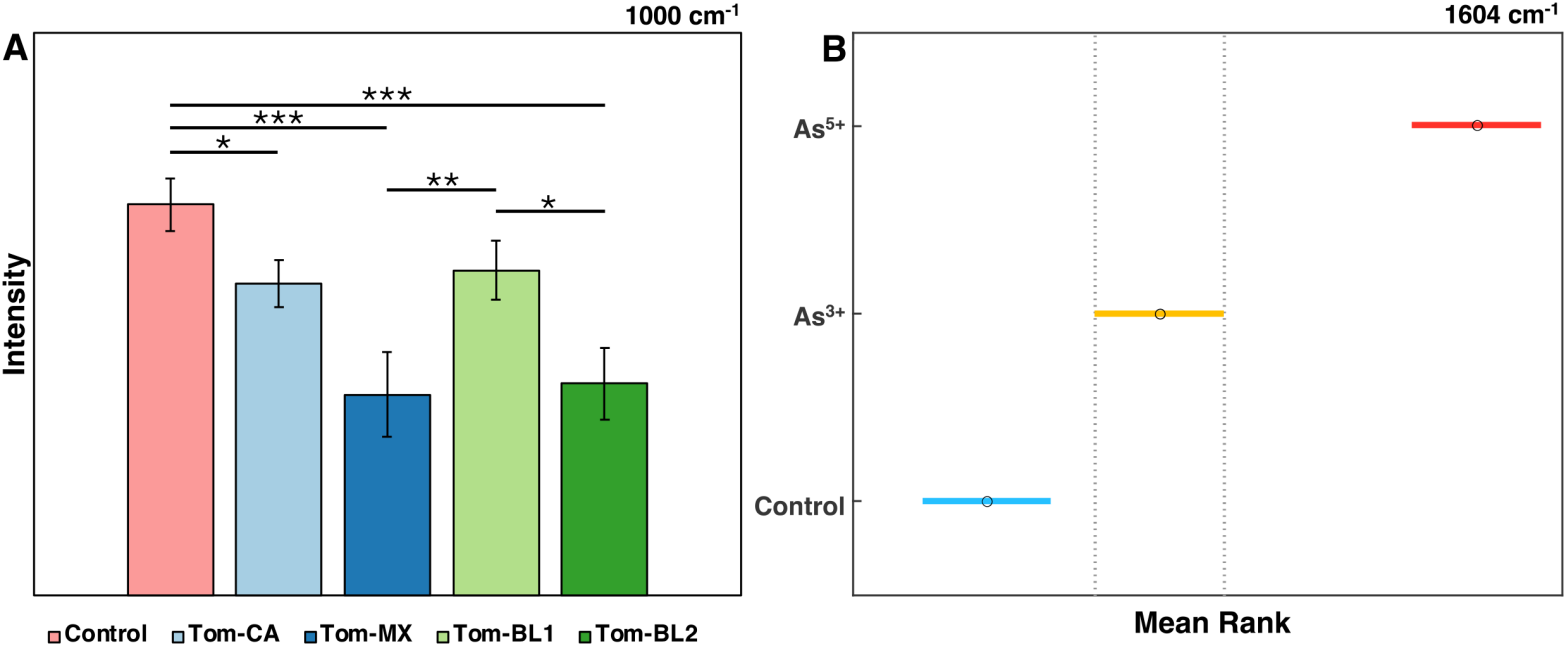
**(A)** Bar graph of Raman intensities at the 1000 cm^-1^ peak by TSWV isolate. * is P ≤ 0.05, ** is P ≤ 0.01, and *** is P ≤ 0.001. **(B)** 95% confidence intervals for the mean rank of intensities at the 1604 cm^-1^ peak by arsenic condition.

The results of these analyses can be visualized in several ways, depending on what is wished to be conveyed. Bar graphs are particularly effective at illustrating statistical differences between groups, especially when many groups clutter the Raman spectral plot (**Figure 3A**). For example, bar graph analysis of the 1000 cm^−1^ peak in tomato plants shows that Tom-MX and Tom-BL2 isolates cause the greatest reduction in carotenoid content. These responses are statistically different from both the control and the Tom-BL1 isolate, but not from each other.

Alternatively, *post-hoc* test results, in this case Dunn’s test, can be depicted directly by showing mean ranks with confidence intervals (**Figure 3B**). This approach provides more direct insight into the statistical results, albeit not conveying information about spectral means. For instance, analysis of the 1604 cm^−1^ peak in rice under arsenic stress shows statistically significant differences between all experimental conditions, indicating the notable increase in phenylpropanoid content as part of the rice stress response.

**Figure 4:**
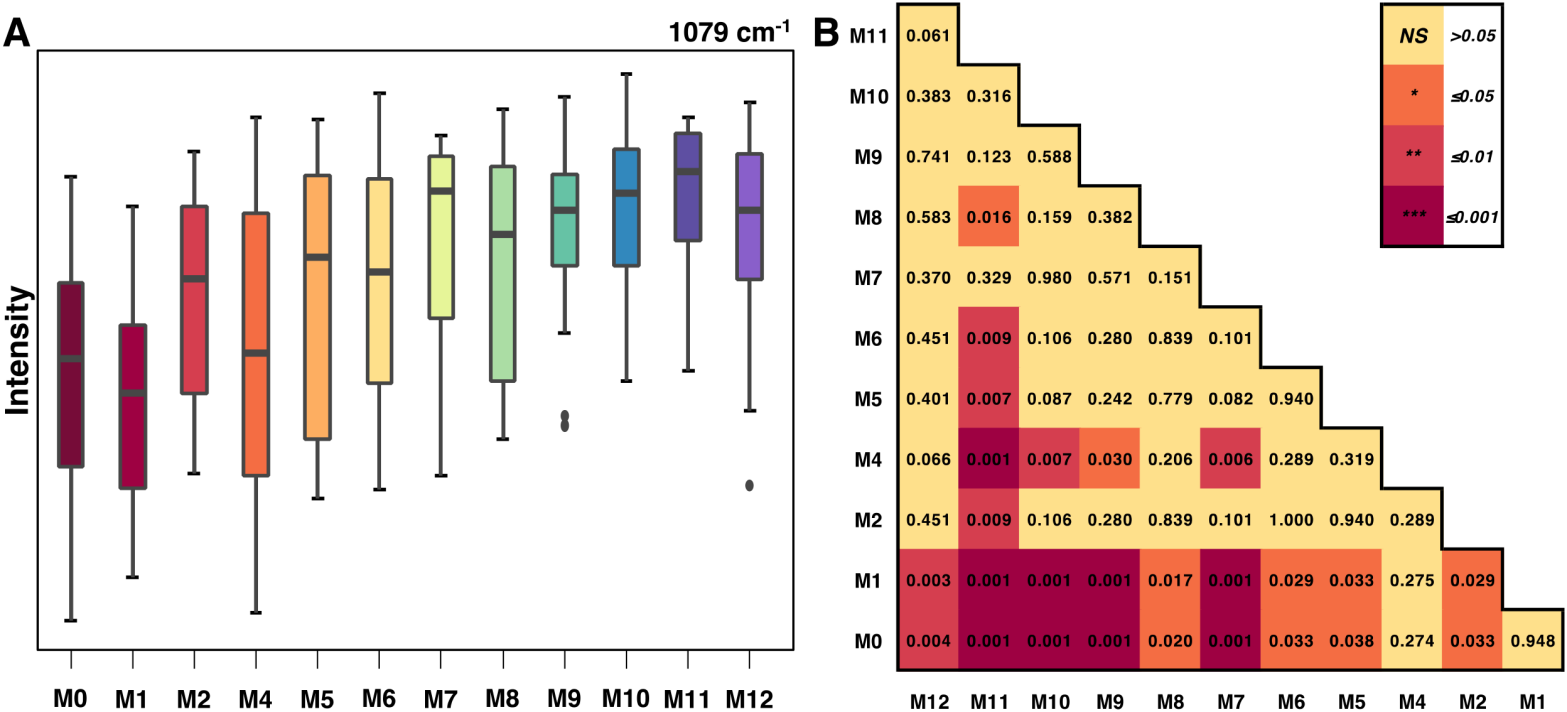
**(A)** Box-and-whiskers plot of Raman intensities at the 1079 cm-1 peak by mice age and **(B)** the associated significance level matrix.

Particularly in experiments with many groups, box-and-whisker plots can be useful for showing the distribution of spectral data at a peak within each group and identifying outliers. Significance level matrices can complement these plots by quickly summarizing the statistical relationships between groups. For example, a box-and-whisker analysis of the 1079 cm^−1^ peak in aging mice showed a clear increase in peak intensity with age, with the first two months displaying the largest statistical differences from following time points (**Figure 4**).

### 2.3 Peak shifting

In some experiments, Raman peaks may shift in response to a perturbation, often signaling structural or environmental changes in a particular molecule or moiety. For example, graphene is commonly characterized by observing shifts in the G-band around 1583 cm^−1^, which corresponds to C-C stretching. Blue shifts in this peak can indicate changes in graphene layer thickness (Park et al., 2009; Tang et al., 2010).

**Figure 5:**
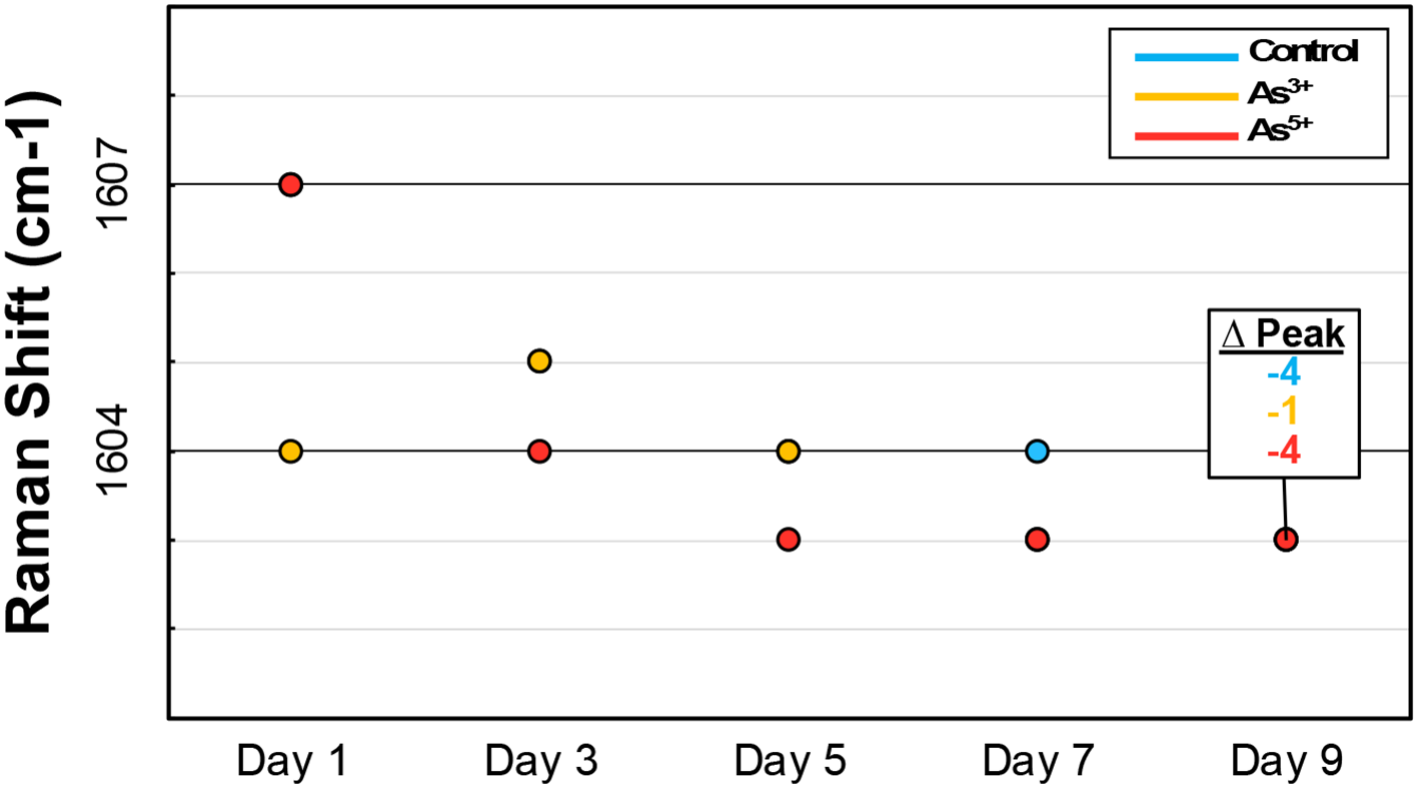
Graph showing peak shifting of the predominant phenylpropanoid peak in rice across the duration of the experiment.

In more complex biological samples, peaks often represent classes of biomolecules rather than individual compounds. Peak shifting indicates a change in the predominant compound within a specific biomolecular class in these instances. For example, phenolics, which typically exhibit peaks around 1600 cm^−1^ and 1630 cm^−1^, consist of over 8,000 distinct compounds with highly variable Raman peaks (Tohge et al., 2013; Jin et al., 2023). Examining the main peak of four hydroxycinnamics, a phenolic subclass involved in lignin production, reveals these slight differences: sinapic acid (1594 cm^−1^), cinnamic acid (1598 cm^−1^), ferulic acid (1601 cm^−1^), and coumaric acid (1605 cm^−1^). Furthermore, in tracking the arsenic stressed rice over nine days, the average location of the primary phenolic peak gradual blue-shifts (**Figure 5**). This likely reflects a change in the predominant phenolic species as the rice matures. As such, these observations provide major context about intra-class molecular dynamics during biological processes.

### 2.4 Peak fitting

Peak fitting is a spectral deconvolution technique that breaks a non-Gaussian peak into its component peaks, typically using mathematical models such as Gaussian, Lorentzian, or Voigt functions. From these, Gaussian functions are most commonly applied. Peak fitting is highly susceptible to overfitting, so it is critical to reference known Raman peak assignments when identify underlying component peaks to ensure accurate results.

**Figure 6:**
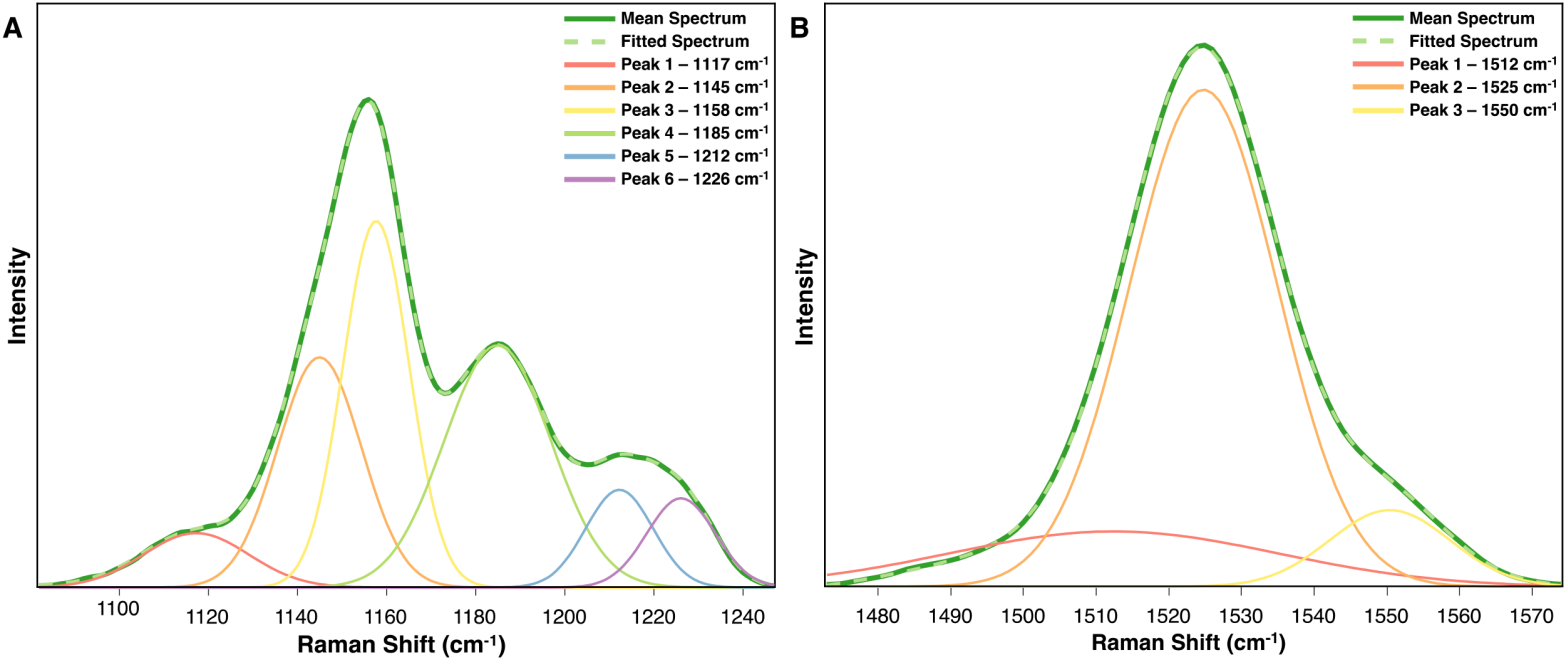
Peak fitted spectra for the carotenoid peaks **(A)** 1155 cm-1 and **(B)** 1525 cm-1 derived from the average rice spectra after 9 days of arsenic stress.

This technique is especially valuable for biological samples, since the vast number of species within a single biomolecular class often contribute to complex peak compositions. For instance, carotenoids comprise over 600 known species (Milani et al., 2017), with their specific structures significantly influencing the Raman peaks. Specifically, functional groups like CH_3_ moieties affect the Raman shift of peaks originating from C-C and C=C stretching, and a carotenoid’s isomeric states can further modify these spectral features (Merlin, 1985). For example, in the average Raman spectrum of arsenic-stressed rice on day 9, minor shoulders on the 1155 cm^−1^ peak suggest the presence of underlying component peak (**Figure 6A**). Peak fitting revealed that this peak consists of two distinct peaks at 1117 cm^−1^ and 1145 cm^−1^. At the 1525 cm^−1^ peak, the primary peak remains, but a smaller, broader peak at 1512 cm^−1^ also influences the spectra throughout the experiment (**Figure 6B**).

By performing peak fitting at various time points, researchers can compare the area under the curve for individual component peaks, providing quantitative information about how specific carotenoid concentrations may be shifting in response to biochemical stress pathways. Coupling peak fitting with full spectrum approaches, like 2D-COS, also enhances the ability to uncover these dynamic composition changes within a sample.

### 2.5 Response characterization

**Figure 7:**
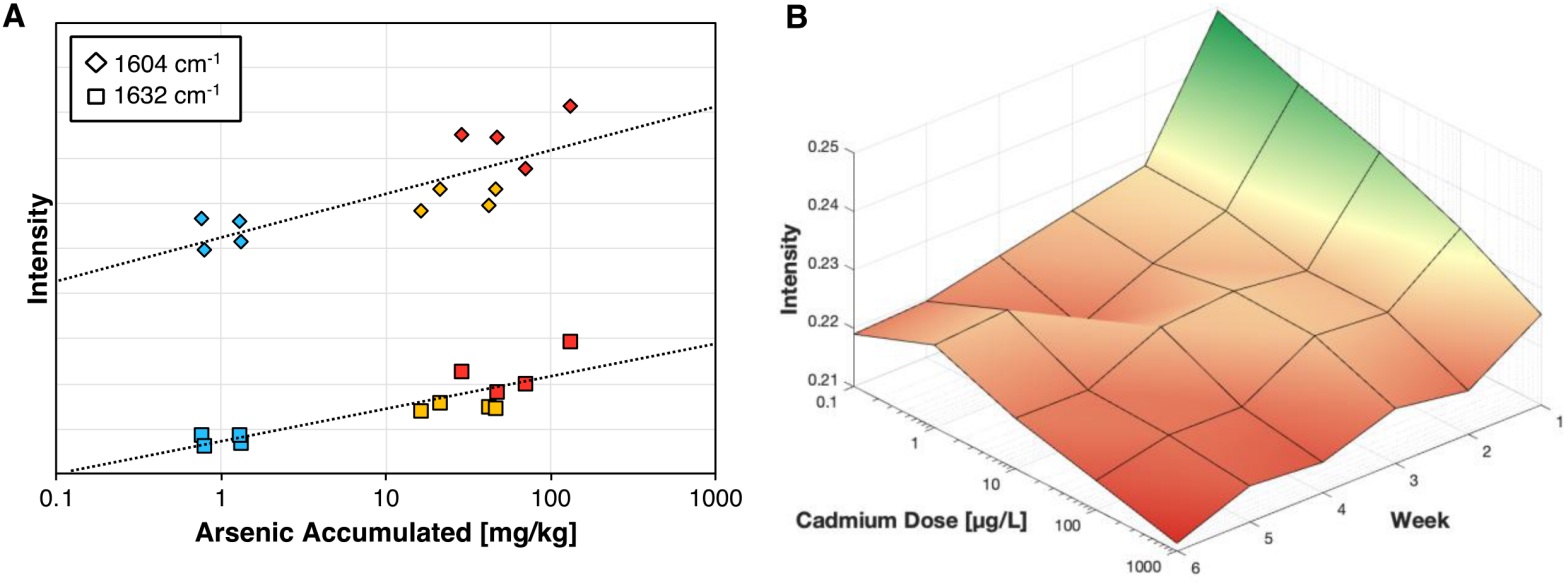
**(A)** Correlation curve for arsenic accumulated in rice tissue vs Raman intensity. **(B)** 3D surface plot for the cadmium stress response at the 1155 cm-1 peak across 6 weeks, with green indicating a higher relative carotenoid concentration.

Response characterization involves analyzing how Raman metrics like peak intensity and area under the curve, change in relation to experimental parameters. This approach helps clarify how the Raman signal changes as a function of the perturbation. For instance, in the study on arsenic-stressed rice, arsenic uptake in the rice tissue was quantified using inductively coupled plasma mass spectrometry (ICP-MS). By plotting arsenic accumulation against Raman peak intensity, correlation curves are generated. The 1604 cm^−1^ and 1632 cm^−1^ phenolic peaks show strong correlations with arsenic levels, with R² values of 0.6499 and 0.7587, respectively. These results demonstrate that RS can potentially bypass ICP-MS in measuring arsenic accumulation (**Figure 7A**). In a separate experiment, increasing levels of cadmium dosage served as the primary perturbation. By incorporating time as a third axis, a 3D surface plot can be generated to track changes in the 1155 cm^−1^ carotenoid peak over six weeks (**Figure 7B**). The data reveals that cadmium stress causes the sharpest decline in intensity after one week, with peak intensity generally decreasing further as the rice matures. However, the intensity reduction due to cadmium stress is always more pronounced than the natural decline associated with rice maturation.

## 3 Full spectrum analyses

### 3.1 2D correlation spectroscopy

2D-COS is a highly informative spectroscopic technique for examining spectral alterations occurring under experimental perturbations. 2D-COS can discern both the sequence and direction of peak changes occurring, by using two complementary correlation maps: the synchronous and asynchronous spectra (Noda, 2015). These correlation maps collectively provide comprehensive insights into the dynamic biochemical changes taking place and can also reveal mechanistic patterns.

**Figure 8:**
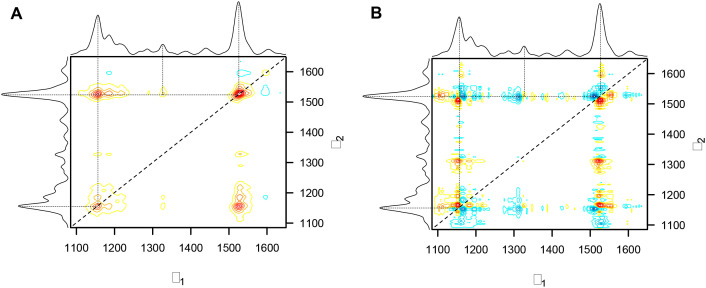
**(A)** Synchronous correlation and **(B)** Asynchronous correlation spectra for arsenic stress across nine days.

Two prominent peaks at 1155 cm^−1^ and 1525 cm^−1^ are positioned along the diagonal in the synchronous spectrum of nine-day arsenic stress experiment, indicating that the two peaks’ intensities change in the same direction as a response to the perturbation (**Figure 8**). These peaks are associated with carotenoids, which play a central role in combating ROS and providing photoprotection. A lower intensity autopeak at 1600 cm^−1^, which is attributed to phenolic compounds, indicates changes related to stress-induced phenolic accumulation. The main cross-peak at the intersection of 1155 cm^−1^ and 1525 cm^−1^ provides additional evidence for their synchronous behavior, while a weak cross-peak at 1326 cm^−1^ and 1525 cm^−1^ suggests interactions between carotenoids and aliphatic signal. From **Figure 1**, all these peaks, except 1600 cm^-1^, are decreasing in intensity, suggesting a degradation of these biomolecules due to arsenic stress.

The asynchronous spectrum is more complicated to interpret but delivers valuable insights about the sequence of the biochemical changes. In this spectrum, a positive cross-peak in both synchronous and asynchronous spectra means that the change in the v_1_ peak happens before the change in the v_2_ peak. Furthermore, the absence of the primary cross-peak (1155 cm^−1^ and 1525 cm^−1^) from the asynchronous spectrum supports the fact that these two carotenoids changes happen simultaneously. Note, that the 1525 cm^−1^ peak is associated with C=C vibrations and the 1155 cm^−1^ peak is associated with C-O-C, C-C bonds, and ring breathing. Mechanistically, this could suggest that carotenoid polyene structures are cleaved concurrently with oxidation and rearrangement of the terminal ring. Peaks found in the asynchronous spectrum but absent in the synchronous spectrum confirm that the sequential changes occur without having a consistent relationship in intensity and point towards independent mechanistic pathways. Notably, the non-Gaussian carotenoid peaks show peak splitting in the asynchronous spectrum, thereby identifying the underlying spectral components at 1150 cm^−1^, 1165 cm^−1^, and 1510 cm^−1^. This could imply that specific carotenoid species are structurally modified or degraded at different rates, most likely as a consequence of arsenic stress-induced peroxidation.

A summary of the asynchronous spectrum is that changes at 1150 cm^−1^ and 1525 cm^−1^ occur prior to changes at 1165 cm^−1^, 1312 cm^−1^, and 1510 cm^−1^. The peak at 1312 cm^−1^ is associated with aliphatic compounds potentially representing changes in membrane lipids, whereas carotenoid-associated peaks at 1150 cm^−1^, 1165 cm^−1^, and 1510 cm^−1^ exhibit a progressive change in the predominant carotenoid species. More clearly stated, the out-of-phase behavior of the underlying carotenoid peaks indicates that arsenic-induced oxidative stress causes preferential degradation or rearrangement of specific carotenoids. While the synchronous spectrum indicates the overall decline in carotenoids is a coordinated process, the asynchronous spectrum indicates a more nuanced development of changes in the underlying peaks, and therefore, highlights distinct mechanistic steps in the plant’s stress response.

### 3.2 PLS-DA model construction

Machine learning algorithms have gained widespread usage in analyzing Raman spectral data because of their ability to reduce dimensionality and extract patterns from complex datasets (Ruiz-Perez et al., 2020). These algorithms include unsupervised techniques, like principal component analysis (PCA), or supervised methods, like partial least squares discriminant analysis (PLS-DA). Unlike unsupervised methods, supervised techniques like PLS-DA are trained on labeled datasets to build predictive models, allowing them to classify new unlabeled data. This feature is especially advantageous for using RS in agricultural diagnostics. PLS-DA excels in classification tasks by maximizing separation between groups while also modeling variance in the predictor variables.

When constructing a PLS-DA model, preprocessing the data can significantly improve model accuracy and reduce bias. Preprocessing should ideally begin with the raw dataset and typically involves three steps: filtering, transformation, and scaling. Filtering processes should be applied first, such as baseline correction, data trimming and removal of nuisance peaks, smoothing and denoising, and transformation including first and higher-order derivatives. Note, each step in preprocessing must be evaluated to avoid overfitting or performance degradation as more transformations do not always result in greater accuracy. For example, when constructing the PLS-DA model to predict mouse age, the best preprocessing combinations were “area normalization + autoscaling” and “1^st^ derivative + median centering” (**Table 1**) In general, less preprocessing and 1^st^ derivatives outperformed more complex models. When screening various combinations of preprocessing, model performance can be quickly assessed using error rate, or the misclassification proportion, and the F1 score, or the harmonic mean of precision and recall.

**Table 1:** Screening results of preprocessing for the PLS-DA model classifying mouse age.

X-processing	Latent Variables	Error Rate	F1 Score
Area Norm., Autoscale	10	0.1275	0.7888
1st Der., Median Center	8	0.1364	0.7708
1st Der., Area Norm., Mean Center	6	0.1376	0.7669
1st Der., Mean Center	8	0.139	0.7662
1st Der., Area Norm., Autoscale	6	0.1428	0.7625
1st Der., Area Norm.	8	0.1405	0.7618
1st Der.	8	0.1425	0.7606
1st Der., Area Norm., Median Center	10	0.1493	0.7491
Area Norm., Mean Center	10	0.1497	0.7447
1st Der., Autoscale	6	0.1571	0.7405
3rd Der., Autoscale	2	0.1569	0.7385
3rd Der., Area Norm., Median Center	6	0.1548	0.7351
3rd Der., Area Norm., Mean Center	6	0.1548	0.7351
Area Norm., Median Center	10	0.1566	0.733
Autoscale	10	0.1601	0.7303
Median Center	10	0.1612	0.7279
3rd Der., Area Norm., Autoscale	2	0.1627	0.727
Mean Center	10	0.1635	0.7241
Area Norm.	10	0.1634	0.7213
2nd Der., Area Norm., Autoscale	4	0.1658	0.7192
3rd Der., Mean Center	10	0.1648	0.7178
3rd Der., Area Norm.	8	0.1645	0.7159
3rd Der.	10	0.1635	0.7145
3rd Der., Median Center	2	0.166	0.7134
2nd Der., Area Norm., Median Center	4	0.1699	0.7109
2nd Der., Area Norm., Mean Center	4	0.1727	0.7077
2nd Der., Autoscale	2	0.1688	0.7038
2nd Der., Area Norm.	2	0.1793	0.6927
2nd Der.	2	0.1717	0.6921
2nd Der., Median Center	4	0.1832	0.6906
2nd Der., Mean Center	4	0.1884	0.684

The preprocessing highlighted red indicates the final preprocessing utilized.

**Figure 9:**
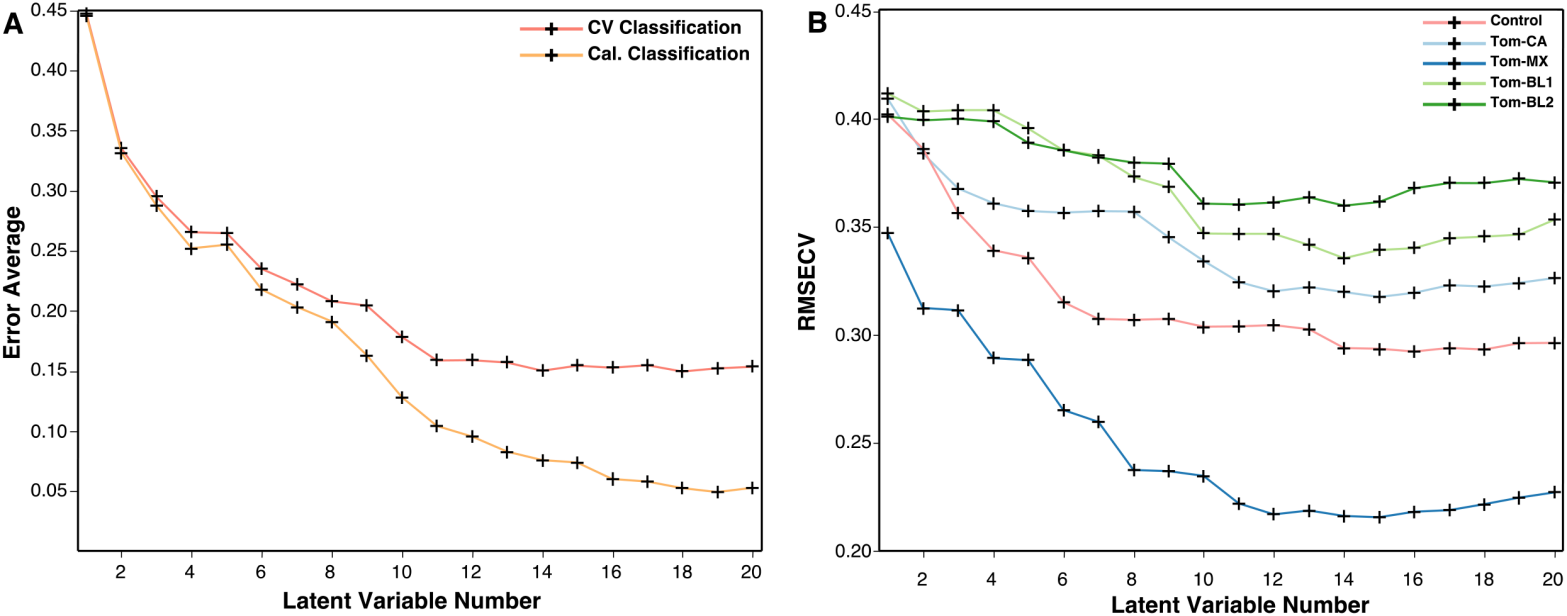
**(A)** Plot of cross-validation and calculated classification error averages and **(B)** root mean square error of cross-validation against the number of latent variables.

After optimizing preprocessing, the best models are then fine-tuned by choosing the optimal number of latent variables (LVs). In building PLS-DA models, the data is converted into LVs, which are analogous to the components in a PCA model (Lasalvia et al., 2022). The distinction is, in PLS-DA, every LV is constructed to maximize covariance between the original data and the classification labels. Increasing the number of LVs captures more variance and generally boost accuracy, but too many LVs can instead result in overfitting, where the model detects noise instead of meaningful patterns, reducing its generalizability, or degree to which the model performs on new data. Conversely, too few LVs can lead to underfitting, in which the model oversimplifies the data and is unable to distinguish between classes, leading to high bias. Data trimming is the first method of reducing these issues, as removing noisy or uninformative regions from the Raman spectrum eliminates the possibility of the model placing any significance on these regions. Then, by tracking the cross-validation classification error and root mean square error of cross-validation (RMSECV), the optimal number of LVs for each model can be determined. These metrics can be used to verify when model accuracy is highest, and model bias is lowest. In the PLS-DA model constructed to discriminate TSWV isolates, cross-validation performance plateaus at around 11 LVs. Similarly, RMSECV is minimized in most classes between 10 and 12 LVs before eventually increasing or plateauing. Based on these observations, 11 LVs were used while building the model to balance accuracy without overfitting (**Figure 9**).

### 3.3 PLS-DA model results

After selecting the appropriate number of LVs, the PLS-DA model generates classification accuracies based on several evaluations. Training results assess how well the model differentiates spectra of labeled classes, while internal validation evaluates how well the model classifies the training spectra that are randomly excluded from the model. Still, since internal validation relies on cross-validation of the training dataset, it can be biased and potentially limit the model’s reliability when applied to new datasets. This limitation is notably important in agricultural diagnostics, where a model trained on a limited, specific dataset may fail when tested on crops grown in unrepresented conditions.

To address this, external validation is essential. Ideally, external validation should involve testing the model on spectra collected from a separate, replicate experiment. If not possible, the dataset can also instead be partitioned into a training set for model construction and internal validation and a validation set for external testing. In the PLS-DA model built to predict TSWV isolates, the model was initially constructed using all spectra for training and internal validation. The model has a slight performance gap between the two but still demonstrates robust predictive capabilities (**Table 2**). The dataset was then partitioned into 70/30, 80/20, and 90/10 training and validation sets. 80/20 is the standard partitioning approach, while testing different splits provides additional insights. A 70/30 split increases the test set size and offers a better assessment of a model’s generalizability. On the other hand, a 90/10 split maximizes training data, making internal validation results more comparable to the fully trained model. This smaller external validation set, however, may not reliably capture spectral variability, leading to skewed and less robust results. In this example, external validation results show that some groups, such as the control and Tom-MX, maintain high true positive rates (TPR) regardless of the partition. Other groups, such as Tom-CA, exhibit a significant decline in TPR, suggesting the model may be overfit for Tom-CA. External validation of Tom-BL2 remained similar to the results of internal validation when using an 80/20 split, however performance dropped drastically for the 90/10 and 70/30 splits. Overall, the external validation results exhibit some inconsistencies between different data splits, implying potential issues with generalizability. The most straightforward solution is to obtain additional spectra from a replicate study to help enhance the model. Furthermore, external validation results can also differ depending on if the data partitioning was done randomly or using an algorithm such as Kennard-Stone or the Onion method, as well as the selection of distance metric, such as using Euclidean or Mahalanobis distances.

**Table 2:** PLS-DA true positivity rate results for the classification of TSWV isolates. Data was partitioned using the Kennard-Stone algorithm based on Euclidean distance.

Isolate	Training Results (%)	Internal Validation (%)	70/30 External Validation (%)	80/20 External Validation (%)	90/10 External Validation (%)
Control	93.3	90.0	81.8	80.0	100
Tom-CA	83.1	75.3	22.6	42.3	54.5
Tom-MX	96.7	90.0	80.0	75.0	100
Tom-BL1	85.6	78.9	79.2	80.0	66.7
Tom-BL2	76.1	68.2	9.4	61.5	38.5

Within each test, model performance can be examined more closely in a confusion matrix, which compares actual class labels to the predicted classifications. Misclassification patterns can then be identified using the matrix, allowing for the calculation of performance measures like TPR. These helps identify model strengths and weaknesses. For instance, in the PLS-DA model for mouse age, most misclassifications are from young adult vs. prime adult mice (**Table 3**). This indicates there is spectral overlap between these classes and means that additional spectra from these classes could be necessary to improve model accuracy. The confusion matrix also enables the calculation of several other performance measures such as positive predictive value (PPV), negative predictive value (NPV), and true negative rate (TNR). These measures offer a more nuanced understanding of the robustness and diagnostic utility of the model. This, in turn, helps identify the model’s strengths and limitations with regards to class differentiation, thereby guiding improvements in experimental design and data collection.

**Table 3:** PLS-DA internal validation confusion matrix for the classification of mouse age.

Actual ClassPredicted Class	TPR (%)	TNR (%)	PPV (%)	NPV (%)	Adolescent (N=19)	Young Adult (N=83)	Prime Adult (N=58)	Middle-Aged (N=80)
Adolescent	100	100	100	100	19	0	0	0
Young Adult	77.1	87.9	77.1	86.2	0	64	14	5
Prime Adult	63.8	85.7	58.7	88.1	0	14	37	12
Middle-Aged	78.8	92.5	84.0	89.7	0	5	7	63

Color intensity correlates with prediction accuracy: the lighter the color the lower is the prediction accuracy. The darker the color, the higher is the prediction accuracy.

In Raman experiments focused on a straightforward diagnostic distinction between healthy and stressed states, binary PLS-DA models can be built. These models compare only two groups, so they usually have higher accuracy due to less classification categories and reduced sensitivity to noise caused by imbalanced class sizes in the training data. When a perturbation is involved, the model’s performance can be evaluated at each perturbation level, providing insights into changes in sensitivity and selectivity. Sensitivity assesses a model’s ability to distinguish stressed spectra from controls, while selectivity assesses its ability to differentiate between two levels or types of stress.

**Figure 10:**
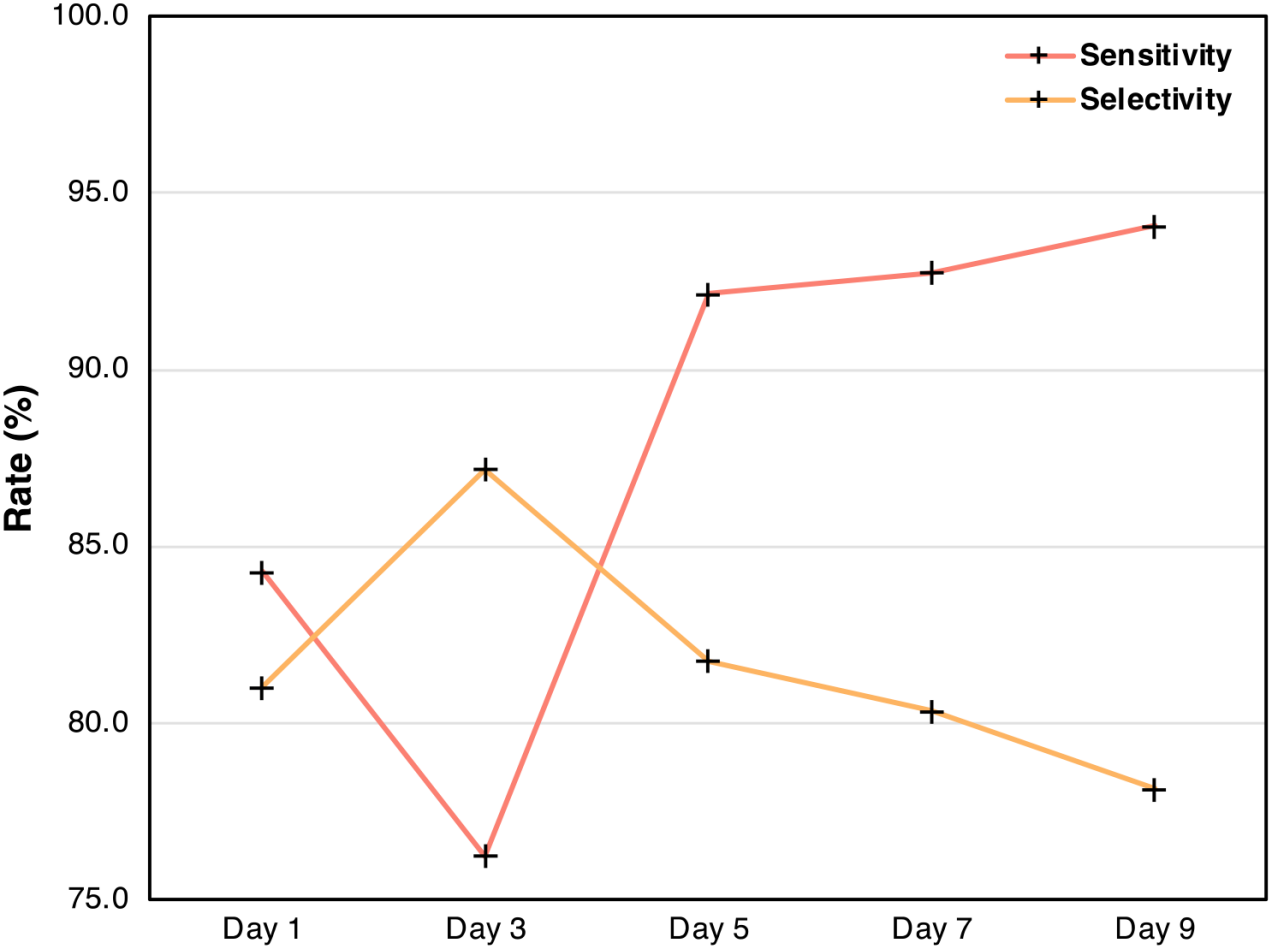
Plot of sensitivity and selectivity from **Table 3** across the duration of the experiment.

In the PLS-DA models differentiating arsenic stress in rice, sensitivity is measured by grouping the two arsenic species, As^5+^ and As^3+^, together and comparing them to the control. Selectivity, on the other hand, is evaluated by testing the model’s ability to differentiate between rice stressed with As^5+^ versus As^3+^. By tracking these metrics over time, the model’s performance across the experiment can be visualized (**Table 4**, **Figure 10**). The results demonstrate that RS becomes more effective at detecting rice crops with high arsenic levels as stress progresses. Inversely, the selectivity trend shows that the model’s ability to distinguish between As^5+^ and As^3+^ worsens over time, likely due to both stressors eventually eliciting a similar biochemical response in the rice crops. These findings suggest that while early detection may better differentiate between forms of arsenic stress, later detection is more reliable for identifying rice crops with elevated levels of arsenic. This demonstrates the added value of conducting perturbation-dependent Raman experiments, which can allow for detailed analyses of temporal trends.

**Table 4:** PLS-DA sensitivity and selectivity results for the classification of arsenic-stressed rice across the duration of the experiment.

Model Metric	Day 1	Day 3	Day 5	Day 7	Day 9
Sensitivity (%)	84.3	76.3	92.1	92.8	93.8
Selectivity (%)	81.0	87.2	81.8	80.3	78.1

### 3.4 PLS-DA model evaluation

Beyond prediction rates, PLS-DA models have several visualization tools to improve understanding of both the model and the data. One example is the cross-validation prediction plot, which shows how strongly the model assigns individual spectra, represented as singular data points, to specific classes. These plots display the thresholds for assignment to a particular class, although final class assignment depends on whether the model uses a strict threshold, where the spectrum to must surpass the threshold for assignment, or a probabilistic approach, where the spectrum is assigned to the class with the highest confidence. Greater separation from the threshold indicates stronger model confidence in classification, resulting in robust model performance.

**Figure 11:**
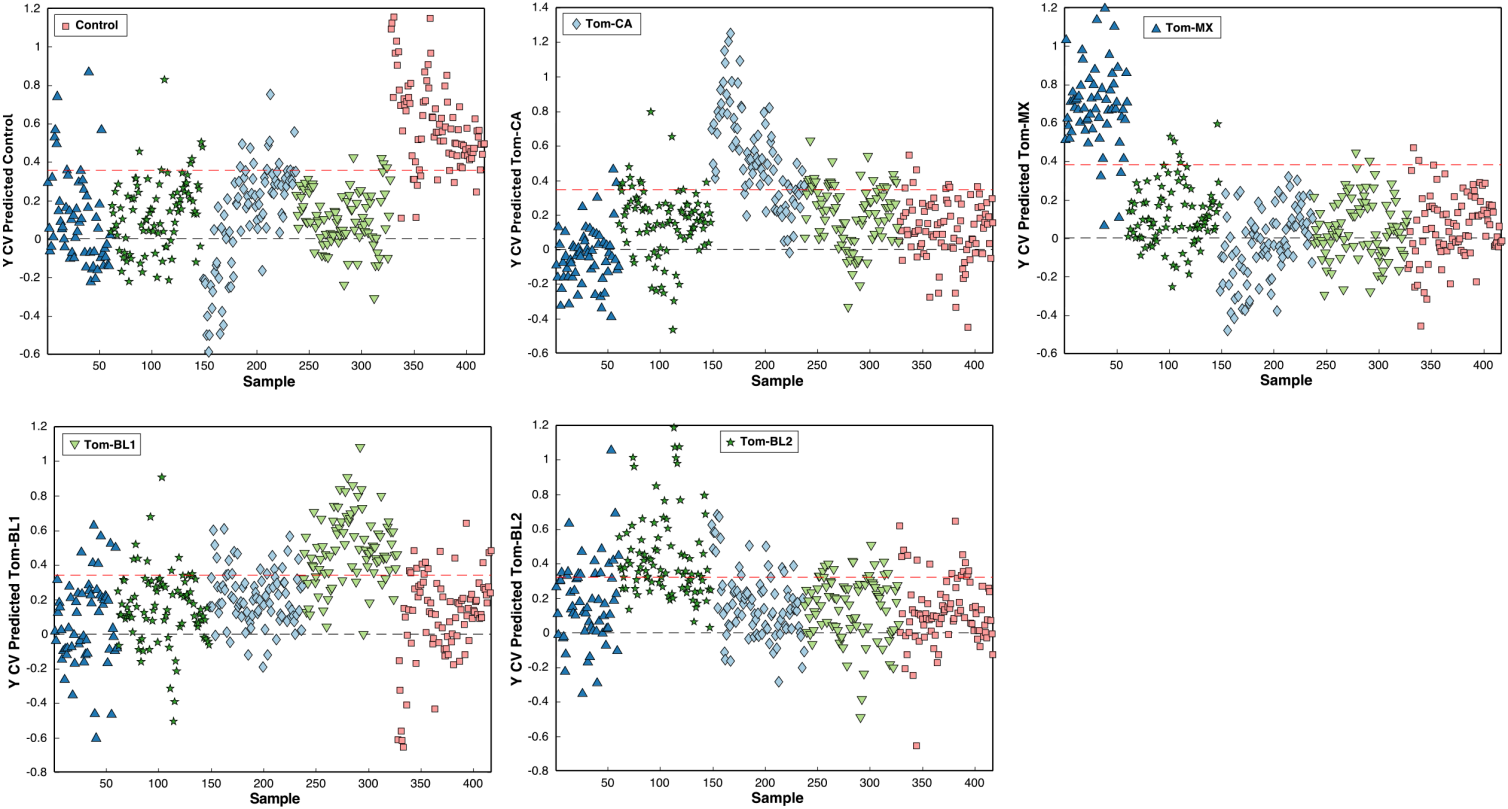
Cross-validation prediction confidence per class per sample.

For instance, in the PLS-DA model for TSWV isolates, spectra for the control and Tom-MX groups show the greatest separation from other classes in their respective plots (**Figure 11**). This corresponds to these groups having the highest training and internal validation results, reflecting not only the model’s accuracy but also its strong confidence in classifying spectra from these classes. Conversely, Tom-CA spectra frequently crossed the thresholds for other classes, consistent with the poor external validation performance for Tom-CA. Similar to confusion matrices, cross-validation plots can also reveal class similarities, such as the overlap between Tom-BL1 and Tom-CA spectra, which were frequently misclassified as one another.

Another 2D visualization tool is the LV scores plot. Similar to a PCA components plot, an LV scores plot shows how spectra cluster by plotting the first two LVs against each other. A high score along an axis indicates that a spectrum aligns strongly with the variance captured by that LV. In an optimal model, confidence intervals for each class should not overlap; however, high model accuracy can still occur even when the first two LVs show class overlap. This often happens in models relying on many LVs, where plotting only the first two does not fully capture class separation. Scores plots can also show the percentage of variance explained by each LV, providing an understanding of the significance of that LV in the classification process.

**Figure 12:**
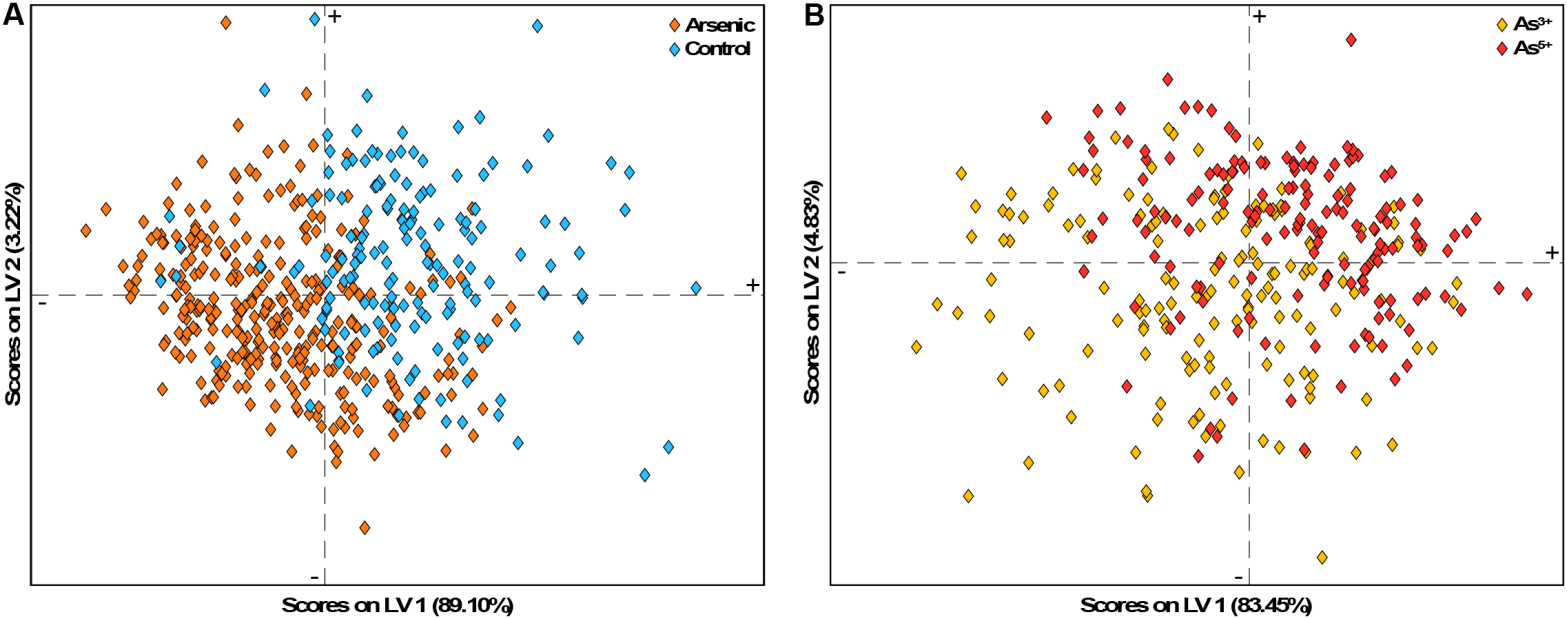
LV scores plot for the PLS-DA models for **(A)** sensitivity and **(B)** selectivity on day 5 of arsenic stress.

In the PLS-DA models for arsenic stress, sensitivity and selectivity scores plots inform that over 80% of the variance is captured by the first LV (**Figure 12**). In the sensitivity plot, most control spectra align closely with the first LV’s pattern. In the selectivity plot, As^5+^ spectra align well with the first LV, but As^3+^ spectra show considerable alignment as well. Even when plotting the second LV, class separation remains unclear, which is consistent with the models’ reliance on an average of six LVs for arsenic stress detection. Lastly, the plots show several outlier spectra whose removal could improve model performance by enhancing class separation. Note, that both cross-validation prediction plots and the scores plots can identify outlier spectra.

One key advantage of PLS-DA over other MLAs is its ability to generate interpretable LV plots, which clarify the contribution of specific features to classification. Within the context of RS, LVs can be visualized as spectra, where deviations from the centerline represent features influencing classification. Positive or negative values indicate how strongly a feature is associated with one class versus another, making this approach particularly informative in binary comparisons. Given the biochemical significance of Raman peaks, parsing the loadings plot can inform which biomolecules are driving class distinctions.

**Figure 13:**
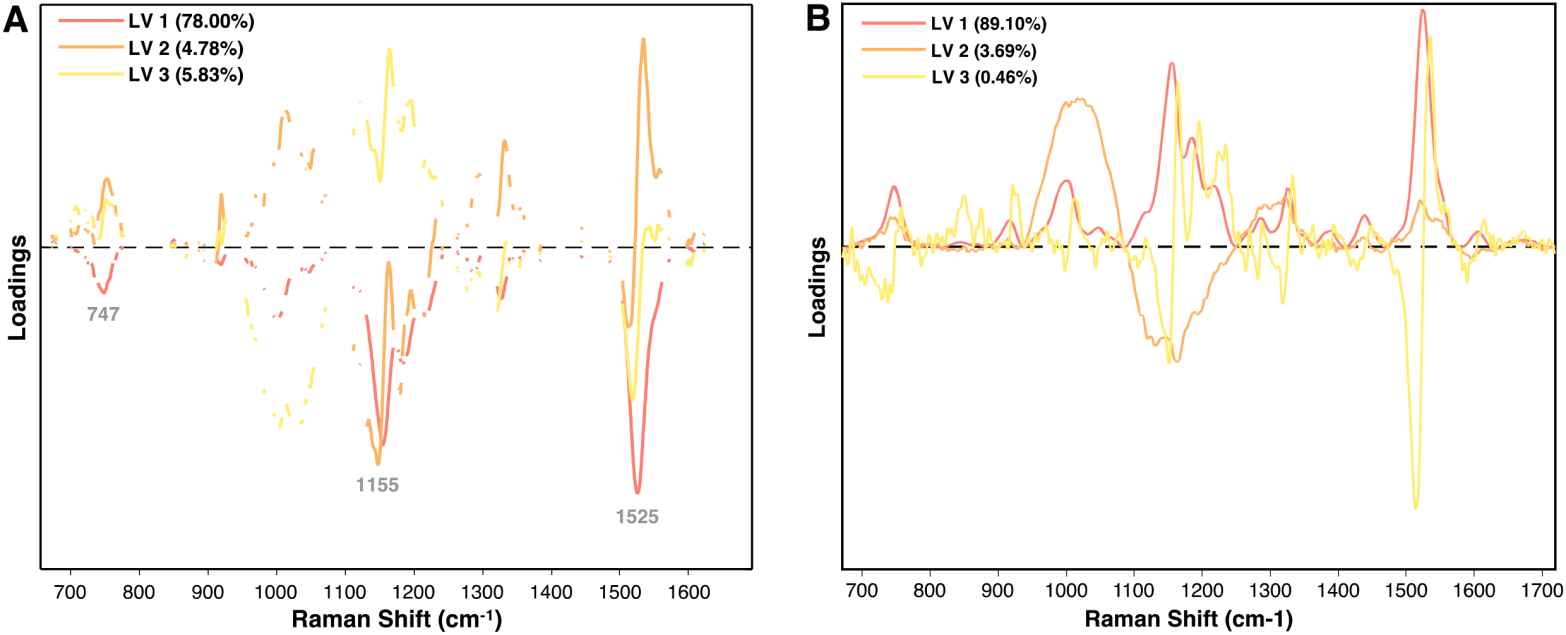
Loadings plot of the first three latent variables for **(A)** the PLS-DA model for TSWV isolates and **(B)** the PLS-DA model for arsenic stress diagnostics at day 5.

This analysis can be further refined using variable selection methods like variable importance in projection (VIP), which assess the significance of each variable within a PLS model. By removing irrelevant variables, VIP improves model performance and prediction accuracy (Xu et al., 2021). In the loadings plots for both TSWV isolates and arsenic stress, carotenoid peaks at 1155 cm^−1^ and 1525 cm^−1^ are the most influential features (**Figure 13**). The TSWV model applied VIP, evident from the gaps in the spectra, whereas the arsenic stress model did not use variable selection, displaying the entire spectra. Notably, phenolic peaks in the 1600–1630 cm^−1^ region, known for their importance in arsenic diagnostics, are largely absent in the TSWV model. Both models are dominated by the first LV, especially the arsenic model, with other LVs having relatively minor influence in classification.

## 4 Limitations and future perspectives

The use of RS in agriculture is set to continue growing, but several challenges must be addressed to fully resolve its potential. Raman data is inherently high-dimensional and complex, making comprehensive statistical analyses somewhat time-consuming and intricate. In addition, RS is a relatively weak phenomenon, and biological samples readily introduce noise from fluorescence and external light. While baseline correction does reduce this background noise, overprocessing the spectra to enhance signal can risk losing critical information (Ryabchykov et al., 2022). Automated baselining methods are nowadays built into Raman instruments and help reduce human bias, but they may also discard meaningful spectral features. This raises concerns about whether manual preprocessing by researchers might better preserve the data quality. Preprocessing methods also substantially impact statistical significance at individual peaks and MLA performance, making careful and unbiased data handling even more important. Even then, instrument-induced spectral variation presents a challenge to reproducibility across studies, highlighting the value of replicating experiments to validate findings (Guo et al., 2020).

The integration of MLAs into RS has been critical in advancing digital agriculture. Although many promising diagnostic models have been developed, their applicability under real-world field conditions often goes unvalidated. Environmental factors influence all biological systems and, thereby, their Raman spectra, so external validation and replication are critical for accurate evaluation of models. Predictive-tree based MLAs, such as extreme gradient boosting discriminant analysis (XGBoost-DA) and random forest, are also gaining traction due to their efficiency, scalability, and ability to handle missing data, enabling the rapid classification of larger, more complex spectral datasets (Amjad et al., 2018; Seifert, 2020; Ranaweera et al., 2021). Still, established techniques like PLS-DA remain invaluable for studying Raman spectra, and more researchers should look past just predictive accuracy, focusing on extracting biological information from latent variables.

Lastly, researchers should prioritize overcoming current limitations by implementing open data practices. Making spectral datasets openly available after publication will enhance reproducibility and accelerate progress in the field of digital agriculture.

As RS continues to gain traction within digital agriculture, a detailed understanding of the information that can be derived is essential. This article has outlined key methods for analyzing Raman spectra and should provide a strong introduction for those new to technology. It covers both discrete peak analyses and full-spectrum approaches, emphasizing their respective strengths and limitations. Understanding the differences in data derivation possible between general comparison studies and perturbation-dependent studies is crucial for effective analysis. With the ongoing progression in data science, emerging techniques are expected to further enhance the utility and impact of RS in agricultural research.

## 5 Methods

The methods for growing rice and tomato crops are described in Juárez et al. (2024b) ^17^,^18^ respectively. B6 mice utilized are described in Juárez et al. (2025) ^11^. In all studies, an Agilent Resolve hand-held Raman spectrophotometer was used to collect spectra from the mice’s abdomens and the crop leaves at 830 nm. Acquisition time was 1 s at a laser power of 495 milliwatts. No spatial offset was used. All spectra were baselined automatically by the Resolve software.

Various software was used in plot-generation and chemometric analysis. The PLS_toolbox (eigenvector Research Inc.) was used in MATLAB to 1) plot and normalize all spectra and 2) build all PLS-DA models and associated plots. Native MATLAB was used to 1) plot Dunn’s test and 2) generate 3D surface plots. R coding language equipped with the corr2d package was used to 1) perform ANOVA and 2) build 2D-COS maps. JASP was used to develop the box-and-whiskers plot. Microsoft Excel was used to 1) create the bar graph, 2) create the significance matrix, 3) plot Raman shift, correlation curves, and sensitivity vs. selectivity. Lastly, peak fitting was done using GRAMS/AI™ Spectroscopy Software.

## Data Availability

The original contributions presented in the study are included in the article/Supplementary Material. Further inquiries can be directed to the corresponding author/s.
